# A case study on strains of Buša cattle structured into a metapopulation to show the potential for use of single-nucleotide polymorphism genotyping in the management of small, cross-border populations of livestock breeds and varieties

**DOI:** 10.3389/fgene.2015.00073

**Published:** 2015-03-05

**Authors:** Elli T. Broxham, Waltraud Kugler, Ivica Medugorac

**Affiliations:** ^1^SAVE FoundationSt. Gallen, Switzerland; ^2^Chair of Animal Genetics and Husbandry, Faculty of Veterinary Medicine, Ludwig-Maximilians-University MunichMunich, Germany

**Keywords:** cross-border, small populations, metapopulations, cattle, Balkans

For over 20 years, SAVE Foundation has concerned itself with the conservation of European livestock breeds. Often, this work has occurred in small populations of cross-border breeds. Livestock has developed over centuries of selection by farmers. The selection has been based on the ecological, climatic, and economic conditions of the local environments. One of these factors, the economic, is affected by the political situation of the country. Country borders as well as human populations have changed many times over the centuries. In contrast, the ecological and climate conditions have generally remained more stable. This means that highly similar locally adapted breeds can often be found both sides of current political borders and are generally grouped within the geo-ecological regions they developed in Druml et al. (2007).

Progressive displacement crossing by highly selected cosmopolitan breeds caused additional fragmentation of locally well-adapted but, in current economic environments, less successful breeds. Remnants from these formerly large populations may not amount to enough stock to make up a nucleus herd for conservation breeding within each single country. However, together with highly similar strains in bordering countries, there may be enough animals to create a combined gene pool and to conserve the locally adapted breed. Up to now, strategies for implementing this conservation method have relied heavily on historical and phenotypical information. Many projects conducted by SAVE over the years have been based on research into historical literature and data, field trips and discussions with experts with local knowledge. The high cost of genotyping combined with the relatively low usefulness of the results has meant that many projects carried out by SAVE and its partners have not used genotyping as a basis for conservation. The advent of SNP genotyping has changed this situation. The relatively low cost for genome-wide genotyping led to a substantial new situation where a higher level of accuracy can be brought into projects without creating a massive financial burden.

An example of this in action is the *BushaLive* project, funded under the “*Funding Strategy for the Implementation of the Global Plan of Action for Animal Genetic Resources.*” The *BushaLive* project targets the autochthonous Buša (also written as Busha) cattle breed of the Balkans. This breed survives in small, highly endangered, sub-populations that are additionally separated from each other due to the break-up of Yugoslavia into smaller states. The Buša is the collective term for small and robust shorthorn (brachyceros) cattle of the Balkans. Residuals from this formerly large Population are currently present in Albania, Bosnia and Herzegovina, Bulgaria, Croatia, Greece, Kosovo^1^, Montenegro, Serbia and The Former Yugoslav Republic of Macedonia. Therefore, it is a typical example of the situation of cross-border breeds (SAVE Foundation, 2014).

The Buša is hardy and well-adapted to extensive farming in challenging environments with low managerial input. The Buša, with their withers' height at around 100 cm, show high fertility and modest production in harsh environments (Kompan et al., 2008). It is an important part of the local identity, but will be lost if conservation measures are not put in place to protect it. Stakeholders across the various nationalities and religions present in the Balkans share a common willingness to collaborate in conserving the breed.

Within the first phase of the *BushaLive* project blood samples have been taken from 254 animals in Albania, Bosnia and Herzegovina, Bulgaria, Croatia, Kosovo (see Footnote 1), Montenegro, Serbia, and The Former Yugoslav Republic of Macedonia. A minimum of 20 samples of possibly unrelated animals per country and strain was requested for the study. To obtain unbiased estimates of the diversity parameters, the population history, genetic differentiation, and the degree of admixture in distinct Buša strains we performed the genome-wide SNP genotyping (the BovineSNP50 BeadChip). To assess admixture and diversity parameters, genotypes of eight reference populations have been included. These populations represent possible sources of admixture as well as having been subject to different levels of artificial selection. Four Buša strains sampled in former studies have also been included. These old samples complement the newly collected material (SAVE Foundation, 2014).

The achieved results provide a framework of future breeding actions and decisions which will be discussed between stakeholders involved in the conservation programme of this fragmented metapopulation (Medugorac et al., 2009). Even if different clusters of strains of Buša cattle are determined as well as different degrees of admixture in some of strains and animals within strains, the final conclusions as well as an estimation of the effective population size will only be possible after completion of all the analyses. However, the results obtained so far show that locally well-adapted strains that have never been intensively managed and differentiated into standardized breeds show large haplotype diversity. This suggests the need for a conservation and recovery strategy that does not rely exclusively on searching for the original native genetic background, but rather on the identification and removal of common introgressed haplotypes (SAVE Foundation, 2014).

Additional to the genotyping further information on each of the sampled animals has been collected via a comprehensive survey targeting their phenotypic characteristics and husbandry systems, as well as the products and services that they provide. This information, together with the genetic data, will be used to provide a basis for the development of a regional strategy for the management of the breed, spanning all stakeholder levels from farmers to governments. The project will also explore the potential for more effective marketing of the breeds' products. The next steps will be the establishment of basic recording systems and support for the development of breeding organizations and common breeding goals. The project will close with a stakeholder workshop for people working at all levels in the conservation of the breed. The event will provide an opportunity to pass on the information gathered and the strategies developed during the project to those who will use them in the future. All the results and data will be published, as they become available, online on: http://agrobiodiversity.net/balkan/topic_network/Bushalive.asp.

## Conflict of interest statement

The authors declare that the research was conducted in the absence of any commercial or financial relationships that could be construed as a potential conflict of interest.

## Footnotes

^1^This designation is without prejudice to positions on status, and is in line with UNSCR 1244 and ICJ advisory opinion on the Kosovo declaration of independence.
